# Dietary lignans and postmenopausal breast cancer risk by oestrogen receptor status: a prospective cohort study of Swedish women

**DOI:** 10.1038/sj.bjc.6604175

**Published:** 2008-01-22

**Authors:** R Suzuki, T Rylander-Rudqvist, S Saji, L Bergkvist, H Adlercreutz, A Wolk

**Affiliations:** 1 Division of Nutritional Epidemiology, The Institute of Environmental Medicine, Karolinska Institute, Stockholm, Sweden; 2 Division of Clinical Trials and Research, Tokyo Metropolitan Cancer and Infectious Diseases Center, Komagome Hospital, Tokyo, Japan; 3 The Department of Surgery and the Center for Clinical Research, Uppsala University, Central Hospital, Västerås, Sweden; 4 Institute for Preventive Medicine, Nutrition, and Cancer, Folkhälsan Research Center, and Division of Clinical Chemistry, Biomedicum, University of Helsinki, Helsinki, Finland

**Keywords:** breast cancer, oestrogen receptor, progesterone receptor, dietary lignans, risk

## Abstract

Among the 51 823 postmenopausal women in the Swedish Mammography Cohort, we investigated breast cancer risk in relation to the FFQ-based estimated lignan intake by oestrogen receptor (ER) and progesterone receptor (PR) subtypes. A significant 17% risk reduction for breast cancer overall in the high lignan quartile was observed, especially among PMH user (*P*_interaction_<0.010), but no heterogeneity across ER/PR subtypes.

Plant lignans, a major type of phytoestrogens in Nordic countries, are mainly present in cereals, fruit, and vegetables (Adlercreutz, 1998a, 1998b) and are metabolised to mammalian lignans (e.g. enterolactone (ENL)) by the intestinal microflora (Adlercreutz, 2002). Since a preventive action of lignans against breast cancer was suggested (Adlercreutz *et al*, 1982), this has been evaluated *in vitro* (Welshons *et al*, 1987; Hirano *et al*, 1990; Mousavi and Adlercreutz, 1992), *in vivo* (Serraino and Thompson, 1991, 1992) and in clinical studies (Adlercreutz *et al*, 1988, 1991; Phipps *et al*, 1993; Thompson *et al*, 2005). Biological plausibility was discussed in a recent review (Adlercreutz, 2007). Hormone-dependent (Adlercreutz *et al*, 1992, 1993) and other mechanisms (Hirano *et al*, 1990; Kitts *et al*, 1999; Mäkelä *et al*, 1999; Prasad, 2000; Rickard *et al*, 2000) have been suggested. Six prospective (den Tonkelaar *et al*, 2001; Keinan-Boker *et al*, 2004; Kilkkinen *et al*, 2004; Olsen *et al*, 2004; Touillaud *et al*, 2007; Verheus *et al*, 2007) and six case–control studies (Pietinen *et al*, 2001; Dai *et al*, 2002; McCann *et al*, 2002, 2004, 2006; Fink *et al*, 2007) have evaluated the issue among postmenopausal women. Of these, only four considered oestrogen and progesterone receptor status of tumours (ER/PR) (den Tonkelaar *et al*, 2001; Olsen *et al*, 2004; McCann *et al*, 2006; Touillaud *et al*, 2007). We therefore examined the issue in a large population-based cohort study with stratification by family history of breast cancer, level of alcohol intake, body mass index, and use of postmenopausal hormone (PMH).

## MATERIALS AND METHODS

The Swedish Mammography Cohort (SMC) was described previously (Wolk *et al*, 1998; Suzuki *et al*, 2006). It was established in 1987–90 that all women in Västmanland who were born in 1917–48, and in Uppsala born in 1914–48, were invited. A total of 66 651 women completed a questionnaire including diet. In 1997, a second questionnaire was sent to all cohort members. We excluded those with missing or incorrect data, with previous cancer (except non-melanoma skin cancer), who were not post-menopausal and who were 70+ years old at baseline leaving a cohort of 51 823 women. The information on diet was collected through self-administrated food-frequency questionnaires in 1987 and 1997. Total lignan intake were estimated using published values of following four lignans; secoisolariciresinol, matairesinol, lariciresinol, and pinoresinol (Mazur *et al*, 1996, 1998a, 1998b, 2000; Adlercreutz and Mazur, 1997; Mazur and Adlercreutz, 1998; Valsta *et al*, 2003; Milder *et al*, 2005; Penalvo *et al*, 2005; Schwartz and Sontag, 2006; Thompson *et al*, 2006). Other nutrients were calculated based on the Swedish National Food Administration database (Bergström *et al*, 1991). Cereals (60%), vegetables (27%), and fruits (10%) are the main sources of our lignans. Among a random sample of 137 women from the cohort, the correlation between the FFQ-based estimates of lignan intake and serum ENL levels measured by time-resolved fluoroimmunoassay (Adlercreutz *et al*, 1998) was *r*=0.2 (Spearman's rank). Date of breast cancer diagnosis, death, or migration from the study area were identified by linkage of the cohort through the Swedish Registration System. Information about receptor status of breast tumours, measured by an Abbott immunoassay (Pousette *et al*, 1986) and an immunohistochemical method, was obtained from Uppsala University Hospital and the Regional Oncology Centre. The study was approved by the Regional Ethics Committee at the Uppsala University Hospital and Karolinska Institute. We used time-dependent multivariate Cox proportional hazards regression model to estimate hazard rate ratios and 95% confidence intervals with age as the time scale (Korn *et al*, 1997). We subdivided lignan intakes into four categories based on approximate quartiles. Trend tests were conducted by using the median value for each category of lignans as a continuous variable. Heterogeneity in the results between the ER+PR+ and other subtypes was evaluated using the Wald statistic (Liao, 2004). *P*-value for interaction was evaluated by a likelihood ratio test. Analyses were performed by SAS system, version 9.1 (SAS Institute, Cary, NC, USA). Statistical tests were two-sided, and significance levels defined as *P*<0.05.

## RESULTS

Among 51 823 women with an average 8.3-year follow-up, 1284 invasive breast cancer cases were diagnosed, with details of ER/PR status available for 1188 cases. Of these, 716 were ER+PR+, 279 ER+PR−, 50 ER−PR+, and 143 ER−PR− tumours. Women with high lignan intake tended to be older, have more education and have greater use of PMH (Table 1).

Overall, we observed a statistically significant inverse association between lignan intake and breast cancer risk (Table 2). Compared to women in the lowest quartile (<712 *μ*g day^−1^), the multivariable adjusted relative risks (RR) for the highest quartile (⩾1036 *μ*g day^−1^) were 0.83 (95% confidence interval=0.70–0.97; *P*_trend_=0.042) for overall, 0.86 (0.69–1.08) for ER+PR+, 0.77 (0.54–1.09) for ER+PR−, 0.92(0.56–1.52) for ER−PR−. There was no evidence for heterogeneity in the results between the ER+PR+ and other subtypes (all *P*_heterogeneities_⩾0.65).

In the full adjusted analysis stratified by family history of breast cancer, by levels of alcohol intake and by body mass index (<25 or ⩾25 kg/m^2^), there was no evidence for interaction with lignans in relation to overall risk or of any subtype; all *P*_values for trends_ were>0.60 and all *P*_*values for interaction*_>0.35. We also observed a significant inverse association of lignans with overall risk among PMH ever-users; the multivariable adjusted RR for the highest quartile of intake compared to the lowest was 46% lower (*P*_trend_=<0.0001; Table 3). In contrast, among PMH never-users, no association was observed (*P*_interaction_=0.01). The observed interaction for PMH use seemed to be confined to ER+PR+ tumors (*P*_interaction_=0.016). There was no heterogeneity in the results between ER+PR+ and other tumors (all *P*_heterogeneity_ ⩾0.21). Lignans were positively correlated with intake of fruits and vegetables (*r*=0.4) and of cereal, fruit and vegetable fibre (*r*=0.7, 0.2 and 0.4, respectively). After adjusting for these factors, the result for lignans was slightly attenuated but still significant among PMH user (Table 3).

## DISCUSSION

In this large population-based prospective cohort of postmenopausal women, we observed a significant inverse association between lignan intake and overall breast cancer risk, especially among PMH user. There was no evidence of heterogeneity across ER/PR tumours. These results are similar to our previous study with a significant inverse association between cereal fibre and breast cancer risk among PMH users (Suzuki *et al*, 2008). The estimated lignan intake was correlated with cereal fibre (*r*=0.7) but after adjusting for specific fibres, the association among PMH users was still significant. This inverse association agrees with two previous studies among postmenopausal women (Fink *et al*, 2007; Touillaud *et al*, 2007). Non-significant inverse associations (Pietinen *et al*, 2001; Dai *et al*, 2002; McCann *et al*, 2002, 2004; Keinan-Boker *et al*, 2004; Olsen *et al*, 2004; Verheus *et al*, 2007) and no association (den Tonkelaar *et al*, 2001; Kilkkinen *et al*, 2004; McCann *et al*, 2006) have also been reported.

An inverse association of lignans with risk has been reported among premenopausal women (Dai *et al*, 2002; McCann *et al*, 2002, 2004, 2006; Linseisen *et al*, 2004; Piller *et al*, 2006a), among women with palpable cysts (Boccardo *et al*, 2004), and high epidermal growth factor concentrations (Boccardo *et al*, 2003), and among those carrying the A2 allele of *CYP17* (McCann *et al*, 2002; Piller *et al*, 2006b) possibly associated with increased levels of endogenous hormone (Haiman *et al*, 1999). Given these findings, an inverse relation of risk with lignans is probable in subgroups of women with high circulating oestrogen level just as discussed with regard to isoflavone (Glazier and Bowman, 2001). The possible biological mechanism is not clear, but *in vitro* studies also showed that lignan ENL in the presence of oestrogens suppressed the oestrogen-induced proliferation in MCF-7 breast cancer cell (Mousavi and Adlercreutz, 1992) and stimulated the synthesis of sex hormone-binding globulin in liver cells (Adlercreutz *et al*, 1992).

The lack of association among overweight women may be due to the relatively high circulating oestrogen levels from PMH use having a stronger effect than the endogenous oestrogens formed in peripheral tissues (Cleland *et al*, 1985; Jurgens *et al*, 1992; Hankinson *et al*, 1998). Compared to lean women, obese women tend to have a lower prevalence of PMH use (Suzuki *et al*, 2006) and lower level of plasma ENL (Kilkkinen *et al*, 2001; Johnsen *et al*, 2004). Body fat might attenuate the effect of lignans by suppressing intestinal microflora activity (Nishizawa *et al*, 1988), or trapping ENL (Johnsen *et al*, 2004).

Our finding for ER+PR+ tumours among PMH users partly agrees with a prospective study (Touillaud *et al*, 2007), though these results were not confined to PMH users. No association was reported in two prospective studies (den Tonkelaar *et al*, 2001; Olsen *et al*, 2004) and a case–control study (McCann *et al*, 2006). Some nutrient misclassification and individual variation in intestinal microflora, as well as the lack of detailed information about PMH use are all relevant. Lignan estimates were not highly correlated with plasma ENL, but the observed correlation was comparable to those reported previously (Kilkkinen *et al*, 2003; Hedelin *et al*, 2006). In prospective cohort design, this misclassification of exposure tends to be nondifferential which may attenuate the observed association toward null. Further studies need to elucidate this issue with taking the circulating level of oestrogens into consideration.

## Figures and Tables

**Table 1:** Age-standardised^a^ characteristics of risk factors for breast cancer according to the levels of lignan intake among 51 823 postmenopausal women in the Swedish Mammography Cohort^b^

	**Quartiles of estimated total lignan intake, *μ*g day^−1^**			
	**Q1**	**Q2**	**Q3**	**Q4**
	**<712**	**712–866**	**867–1035**	**⩾1036**
**Characteristics**	***n*=12 730 (24.6%)**	***n*=13 030 (25.1%)**	***n*=13 011 (25.1%)**	***n*=13 052 (25.2%)**
Intake of lignans, *μ*g day^−1^, median	613.6	791.8	942.7	1175.1
Age at entry, years, mean (s.d.)	59.1 (8.1)	59.1 (7.9)	59.6 (7.8)	60.6 (7.7)
Age at menarche, years, mean (s.d.)	13.2 (1.3)	13.2 (1.2)	13.2 (1.2)	13.2 (1.3)
Age at first birth, years, mean (s.d.)	23.9 (4.5)	24.2 (4.6)	24.2 (4.5)	24.1 (4.4)
Body mass index, kg m^−2^, mean (s.d.)	25.2 (4.1)	25.2 (3.9)	25.1 (3.9)	25.1 (4.0)
Number of children, *n*, mean (s.d.)	2.1 (1.3)	2.2 (1.2)	2.1 (1.2)	2.1 (1.3)
Age at menopause, years, mean (s.d.)	50.6 (4.9)	50.8 (4.8)	50.9 (4.6)	50.8 (4.8)
⩾12 years of education, %	8.1	10.1	11.1	12.4
Ever use of oral contraceptives, %	53.5	54.3	54.8	54.2
Ever use of postmenopausal hormones, %	42.1	44.7	46.6	44.8
Family history of breast cancer, %^c^	7.8	8.5	8.2	8.0
Total energy intake, kcal day^−1^, mean (s.d.)	1532 (447)	1604 (428)	1616 (421)	1628 (465)
Total fat intake, g day^−1^, mean (s.d.)	56.0 (8.3)	53.1 (7.5)	50.9 (7.6)	47.7 (8.3)
Alcohol intake, ethanol g day^−1^, mean (s.d.)	3.2 (5.1)	3.6 (5.4)	3.5 (4.5)	3.1 (6.1)

s.d.=standard deviation.

a Age-standardised to the distribution of person–time of follow-up among all study participants.

b Based on the information at 1987 and 1997.

c Breast cancer in mother, sister, or daughter.

**Table 2:** Relative risks (RRs) and 95% confidence intervals for the association between FFQ-based estimated intake of lignans and postmenopausal breast cancer risk by receptor-defined subtype among 51 823 postmenopausal women in the Swedish Mammography Cohort

		**Quartiles of estimated total lignan intake, *μ*g day^−1^**					
**Categories for quartile**		**Q1**	**Q2**	**Q3**	**Q4**		
**Lignan intake, *μ*g day^−1^**	**No. of cases**	**<712**	**712–866**	**867–1035**	**⩾1036**	***P* a**	***P* b**
No of person-year		101 994	105 399	107 791	115 147		
							
*All invasive tumours*							
Age-adjusted RR	1284	1.00	0.86 (0.74–1.00)	0.87 (0.74–1.01)	0.86 (0.74–1.00)	0.09	
Multivariable-adjusted RR^c^	1284	1.00	0.83 (0.71-0.97)	0.83 (0.70-0.97)	0.83 (0.70-0.97)	0.042	
							
*ER*+*PR*+*tumours*							
Age-adjusted RR	716	1.00	0.82 (0.66-1.01)	0.90 (0.73-1.10)	0.89 (0.72-1.09)	0.44	
Multivariable-adjusted RR^c^	716	1.00	0.79 (0.63–0.97)	0.86 (0.69–1.06)	0.86 (0.69–1.08)	0.35	
							
*ER*+*PR*−*tumours*							
Age-adjusted RR	279	1.00	0.81 (0.59-1.12)	0.67 (0.48-0.94)	0.77 (0.56-1.07)	0.09	
Multivariable-adjusted RR^c^	279	1.00	0.77 (0.56–1.07)	0.64 (0.45–0.90)	0.77 (0.54–1.09)	0.12	0.65
							
*ER*−*PR*−*tumours*							
Age-adjusted RR	143	1.00	0.85 (0.53-1.36)	0.93 (0.59-1.46)	0.87 (0.55-1.38)	0.66	
Multivariable-adjusted RR^c^	143	1.00	0.87 (0.54–1.40)	0.96 (0.60–1.54)	0.92 (0.56–1.52)	0.86	0.99

ER, oestrogen receptor; PR, progesterone receptor.

a Two sided *P*-values for trend were calculated using the Wald statistics using the median values for each category of intake of lignan as continuous variable.

b *P*-values (two-sided) for heterogeneity from the Wald test compared with four pairs of *β*-coefficients of ER+PR+tumours.

c Multivariable Cox proportional harzard models with age as the time-scales were adjusted for height (continuous), body mass index (<18.5, 18.5–24.9, 25–29.9, ⩾30 kg m^−2^), education (<12 years of education, ⩾12 years of education), parity (nulliparous, 1–2, ⩾3), age at first birth (nulliparous, <26, 26–30, ⩾31 years), age at menarche (⩽12, 13, ⩾14 years, missing), age at menopause (<51, ⩾51 years), type of menopause (natural, surgery), use of oral contraceptives (ever, never, missing), use of postmenopausal hormones (ever, never, missing), family history of breast cancer among first-degree relatives (yes/no), history of benign breast disease (yes/no), quintiles of total energy intake, quintiles of energy-adjusted total fat intake, and alcohol intake (nondrinkers, <3.4, 3.4–9.9, ⩾10.0 ethanol g day^−1^).

**Table 3:** Multivariable relative risks (RRs) and 95% confidence intervals (CI) for the association between total lignan intake and all postmenopausal breast cancer risk among 41 795 postmenopausal women^a^ in the Swedish Mammography Cohort with stratified by use of PMH

		**Quartiles of estimated total lignan intake, *μ*g day^−1^**									
		**Q1**		**Q2**		**Q3**		**Q4**			
	**No of cases**	**No**	**Ref.**	**No**	**RR (95%CI)**	**No**	**RR (95%CI)**	**No**	**RR (95%CI)**	***P*_trend_ b**	***P*_int_ c**
*Use of PMH* d											
Ever	446	117	1.00	133	0.85 (0.66–1.10)	119	0.75 (0.57–0.98)	77	0.54 (0.39–0.73)	<0.0001	<0.01
Never	528	139	1.00	109	0.72 (0.55–0.92)	127	0.85 (0.66–1.09)	153	0.97 (0.76–1.25)	0.69	
											
*Use of PMH* e											
Ever	446	117	1.00	133	0.90 (0.68–1.19)	119	0.83 (0.59–1.17)	77	0.64 (0.42–0.99)	0.042	0.010
Never	528	139	1.00	109	0.80 (0.61–1.06)	127	1.06 (0.78–1.44)	153	1.26 (0.88–1.80)	0.07	

a Among 41 795 postmenopausal women with complete information for PMH use in the Swedish Mammography Cohort.

b Two-sided *P*-values for trend were calculated using the median values for each category of dietary lignan intake as continuous variable.

c Two-sided *P*-values for interaction were calculated based on −2 log likelihood test based on the model.

d Multivariable-adjusted RR adjusted for age (the time-scale), height (continuous), education (<12 years of education, ⩾12 years of education), parity (nulliparous, 1–2, ⩾3), age at first birth (nulliparous, <26, 26–30, ⩾31 years), age at menarche (⩽12, 13, ⩾14 years, missing), age at menopause (<51, ⩾51 years), type of menopause (natural, surgery), use of oral contraceptives (ever, never, missing), use of postmenopausal hormones (ever, never, missing), total energy intake (quintiles), energy adjusted total fat intake (quintiles), alcohol intake (nondrinkers, <3.4, 3.4–9.9, ⩾10.0).

e Multivariable-adjusted model as above further adjusted for consumption of fruits and vegetables (quintiles), energy-adjusted dietary fibre intake (quintile; cereal, fruit, and vegetable fibre independently).

## References

[bib1] Adlercreutz H (1998a) Epidemiology of Phytoestrogens. London: Bailliere Tindall 10.1016/s0950-351x(98)80007-4 10384816

[bib2] Adlercreutz H (1998b) Epidemiology of phytoestrogens. Baillieres Clin Endocrinol Metab 12: 605–623 10384816 10.1016/s0950-351x(98)80007-4

[bib3] Adlercreutz H (2002) Phyto-oestrogens and cancer. Lancet Oncol 3: 364–373 12107024 10.1016/s1470-2045(02)00777-5

[bib4] Adlercreutz H (2007) Lignans and human health. Crit Rev Clin Lab Sci 44: 483–525 17943494 10.1080/10408360701612942

[bib5] Adlercreutz H, Bannwart C, Wähälä K, Mäkelä T, Brunow G, Hase T, Arosemena PJ, Kellis Jr JT, Vickery LE (1993) Inhibition of human aromatase by mammalian lignans and isoflavonoid phytoestrogens. J Steroid Biochem Mol Biol 44: 147–153 8382517 10.1016/0960-0760(93)90022-o

[bib6] Adlercreutz H, Fotsis T, Heikkinen R, Dwyer JT, Woods M, Goldin BR, Gorbach SL (1982) Excretion of the lignans enterolactone and enterodiol and of equol in omnivorous and vegetarian postmenopausal women and in women with breast cancer. Lancet 2: 1295–1299 6128595 10.1016/s0140-6736(82)91507-0

[bib7] Adlercreutz H, Höckerstedt K, Bannwart C, Hämäläinen E, Fotsis T, Bloigu S (1988) Association between dietary fiber, urinary excretion of lignans and isoflavonic phytoestrogens, and plasma non-protein bound sex hormones in relation to breast cancer. In Progress in Cancer Research and Therapy: Hormones and Cancer, Bresciani F, King RJB, Lippman ME, Raynaud J-P (eds) Vol. 35, 409–412. New York: Raven Press, Ltd

[bib8] Adlercreutz H, Honjo H, Higashi A, Fotsis T, Hämäläinen E, Hasegawa T, Okada H (1991) Urinary excretion of lignans and isoflavonoid phytoestrogens in Japanese men and women consuming a traditional Japanese diet. Am J Clin Nutr 54: 1093–1100 1659780 10.1093/ajcn/54.6.1093

[bib9] Adlercreutz H, Mazur W (1997) Phyto-oestrogens and Western diseases. Ann Med 29: 95–120 9187225 10.3109/07853899709113696

[bib10] Adlercreutz H, Mousavi Y, Clark J, Höckerstedt K, Hämäläinen E, Wähäla K, Mäkelä T, Hase T (1992) Dietary phytoestrogens and cancer: *in vitro* and *in vivo* studies. J Steroid Biochem Mol Biol 41: 331–337 1314077 10.1016/0960-0760(92)90359-q

[bib11] Adlercreutz H, Wang GJ, Lapcik O, Hampl R, Wähälä K, Mäkelä T, Lusa K, Talme M, Mikola H (1998) Time-resolved fluoroimmunoassay for plasma enterolactone. Anal Biochem 265: 208–215 9882394 10.1006/abio.1998.2886

[bib12] Bergström LKE, Hagman U, Eriksson HB, Bruce Å (1991) The food composition database KOST: the National Food Administration's information system for nutritive values of food. Vår Föda 43: 439–447

[bib13] Boccardo F, Lunardi G, Guglielmini P, Parodi M, Murialdo R, Schettini G, Rubagotti A (2004) Serum enterolactone levels and the risk of breast cancer in women with palpable cysts. Eur J Cancer 40: 84–89 14687793 10.1016/s0959-8049(03)00576-8

[bib14] Boccardo F, Lunardi GL, Petti AR, Rubagotti A (2003) Enterolactone in breast cyst fluid: correlation with EGF and breast cancer risk. Breast Cancer Res Treat 79: 17–23 12779078 10.1023/a:1023356209478

[bib15] Cleland WH, Mendelson CR, Simpson ER (1985) Effects of aging and obesity on aromatase activity of human adipose cells. J Clin Endocrinol Metab 60: 174–177 3964790 10.1210/jcem-60-1-174

[bib16] Dai Q, Franke AA, Jin F, Shu XO, Hebert JR, Custer LJ, Cheng J, Gao YT, Zheng W (2002) Urinary excretion of phytoestrogens and risk of breast cancer among Chinese women in Shanghai. Cancer Epidemiol Biomarkers Prev 11: 815–821 12223424

[bib17] den Tonkelaar I, Keinan-Boker L, Veer PV, Arts CJ, Adlercreutz H, Thijssen JH, Peeters PH (2001) Urinary phytoestrogens and postmenopausal breast cancer risk. Cancer Epidemiol Biomarkers Prev 10: 223–228 11303591

[bib18] Fink BN, Steck SE, Wolff MS, Britton JA, Kabat GC, Schroeder JC, Teitelbaum SL, Neugut AI, Gammon MD (2007) Dietary flavonoid intake and breast cancer risk among women on Long Island. Am J Epidemiol 165: 514–523 17158855 10.1093/aje/kwk033

[bib19] Glazier MG, Bowman MA (2001) A review of the evidence for the use of phytoestrogens as a replacement for traditional estrogen replacement therapy. Arch Intern Med 161: 1161–1172 11343439 10.1001/archinte.161.9.1161

[bib20] Haiman CA, Hankinson SE, Spiegelman D, Colditz GA, Willett WC, Speizer FE, Kelsey KT, Hunter DJ (1999) The relationship between a polymorphism in CYP17 with plasma hormone levels and breast cancer. Cancer Res 59: 1015–1020 10070957

[bib21] Hankinson SE, Willett WC, Manson JE, Colditz GA, Hunter DJ, Spiegelman D, Barbieri RL, Speizer FE (1998) Plasma sex steroid hormone levels and risk of breast cancer in postmenopausal women. J Natl Cancer Inst 90: 1292–1299 9731736 10.1093/jnci/90.17.1292

[bib22] Hedelin M, Klint A, Chang ET, Bellocco R, Johansson JE, Andersson SO, Heinonen SM, Adlercreutz H, Adami HO, Gronberg H, Balter KA (2006) Dietary phytoestrogen, serum enterolactone and risk of prostate cancer: the cancer prostate Sweden study (Sweden). Cancer Causes Control 17: 169–180 16425095 10.1007/s10552-005-0342-2

[bib23] Hirano T, Fukuoka K, Oka K, Naito T, Hosaka K, Mitsuhashi H, Matsumoto Y (1990) Antiproliferative activity of mammalian lignan derivatives against the human breast carcinoma cell line, ZR-75-1. Cancer Invest 8: 595–602 2149833 10.3109/07357909009018926

[bib24] Johnsen NF, Hausner H, Olsen A, Tetens I, Christensen J, Knudsen KE, Overvad K, Tjonneland A (2004) Intake of whole grains and vegetables determines the plasma enterolactone concentration of Danish women. J Nutr 134: 2691–2697 15465768 10.1093/jn/134.10.2691

[bib25] Jurgens Jr RW, Downey LJ, Abernethy WD, Cutler NR, Conrad J (1992) A comparison of circulating hormone levels in postmenopausal women receiving hormone replacement therapy. Am J Obstet Gynecol 167: 459–460 1497051 10.1016/s0002-9378(11)91429-x

[bib26] Keinan-Boker L, van Der Schouw YT, Grobbee DE, Peeters PH (2004) Dietary phytoestrogens and breast cancer risk. Am J Clin Nutr 79: 282–288 14749235 10.1093/ajcn/79.2.282

[bib27] Kilkkinen A, Stumpf K, Pietinen P, Valsta LM, Tapanainen H, Adlercreutz H (2001) Determinants of serum enterolactone concentration. Am J Clin Nutr 73: 1094–1100 11382665 10.1093/ajcn/73.6.1094

[bib28] Kilkkinen A, Valsta LM, Virtamo J, Stumpf K, Adlercreutz H, Pietinen P (2003) Intake of lignans is associated with serum enterolactone concentration in Finnish men and women. J Nutr 133: 1830–1833 12771325 10.1093/jn/133.6.1830

[bib29] Kilkkinen A, Virtamo J, Vartiainen E, Sankila R, Virtanen MJ, Adlercreutz H, Pietinen P (2004) Serum enterolactone concentration is not associated with breast cancer risk in a nested case-control study. Int J Cancer 108: 277–280 14639615 10.1002/ijc.11519

[bib30] Kitts DD, Yuan YV, Wijewickreme AN, Thompson LU (1999) Antioxidant activity of the flaxseed lignan secoisolariciresinol diglycoside and its mammalian lignan metabolites enterodiol and enterolactone. Mol Cell Biochem 202: 91–100 10705999 10.1023/a:1007022329660

[bib31] Korn EL, Graubard BI, Midthune D (1997) Time-to-event analysis of longitudinal follow-up of a survey: choice of the time-scale. Am J Epidemiol 145: 72–80 8982025 10.1093/oxfordjournals.aje.a009034

[bib32] Liao TF (2004) Comparing social groups: Wald Statistics for testing equality among Multiple Logit Models. Int J Comparative Sociology 45: 3–16

[bib33] Linseisen J, Piller R, Hermann S, Chang-Claude J (2004) Dietary phytoestrogen intake and premenopausal breast cancer risk in a German case–control study. Int J Cancer 110: 284–290 15069695 10.1002/ijc.20119

[bib34] Mäkelä S, Strauss L, Saarinen N, Salmi S, Streng T, Joshi S, Santti R (1999) Dietary phytoestrogens—mechanisms of action and possible role in the development of hormonally dependent diseases. In Natural Antioxidants and Anticarcinogens in Nutrition, Health and Disease, Kumpulainen JT, Salonen JT (eds) pp 349–355. London: Royal Society of Chemistry

[bib35] Mazur W, Adlercreutz H (1998) Naturally occurring oestrogens in food. Pure & Appl Chem 70: 1759–1776

[bib36] Mazur W, Fotsis T, Wähälä K, Ojala S, Salakka A, Adlercreutz H (1996) Isotope dilution gas chromatographic-mass spectrometric method for the determination of isoflavonoids, coumestrol, and lignans in food samples. Anal Biochem 233: 169–180 8789715 10.1006/abio.1996.0025

[bib37] Mazur WM, Duke JA, Wähälä K, Rasku S, Adlercreutz H (1998a) Isoflavonoids and lignans in legumes: nutritional and health aspects in humans. J Nutr Biochem 9: 193–200

[bib38] Mazur WM, Uehara M, Wähälä K, Adlercreutz H (2000) Phyto-oestrogen content of berries, and plasma concentrations and urinary excretion of enterolactone after a single strawberry-meal in human subjects. Br J Nutr 83: 381–387 10858696

[bib39] Mazur WM, Wähälä K, Rasku S, Salakka A, Hase T, Adlercreutz H (1998b) Lignan and isoflavonoid concentrations in tea and coffee. Br J Nutr 79: 37–45 9505801 10.1079/bjn19980007

[bib40] McCann SE, Kulkarni S, Trevisan M, Vito D, Nie J, Edge SB, Muti P, Freudenheim JL (2006) Dietary lignan intakes and risk of breast cancer by tumor estrogen receptor status. Breast Cancer Res Treat 99: 309–311 16541305 10.1007/s10549-006-9196-x

[bib41] McCann SE, Moysich KB, Freudenheim JL, Ambrosone CB, Shields PG (2002) The risk of breast cancer associated with dietary lignans differs by CYP17 genotype in women. J Nutr 132: 3036–3041 12368392 10.1093/jn/131.10.3036

[bib42] McCann SE, Muti P, Vito D, Edge SB, Trevisan M, Freudenheim JL (2004) Dietary lignan intakes and risk of pre- and postmenopausal breast cancer. Int J Cancer 111: 440–443 15221974 10.1002/ijc.20262

[bib43] Milder IE, Arts IC, van de Putte B, Venema DP, Hollman PC (2005) Lignan contents of Dutch plant foods: a database including lariciresinol, pinoresinol, secoisolariciresinol and matairesinol. Br J Nutr 93: 393–402 15877880 10.1079/bjn20051371

[bib44] Mousavi Y, Adlercreutz H (1992) Enterolactone and estradiol inhibit each other's proliferative effect on MCF-7 breast cancer cells in culture. J Steroid Biochem Mol Biol 41: 615–619 1562532 10.1016/0960-0760(92)90393-w

[bib45] Nishizawa Y, Imaizumi T, Tanishita H, Yano I, Kawai Y, Mormii H (1988) Relationship of fat deposition and intestinal microflora in VMH rats. Int J Obes 12: 103–110 3384556

[bib46] Olsen A, Knudsen KE, Thomsen BL, Loft S, Stripp C, Overvad K, Moller S, Tjonneland A (2004) Plasma enterolactone and breast cancer incidence by estrogen receptor status. Cancer Epidemiol Biomarkers Prev 13: 2084–2089 15598765

[bib47] Penalvo JL, Haajanen KM, Botting N, Adlercreutz H (2005) Quantification of lignans in food using isotope dilution gas chromatography/mass spectrometry. J Agric Food Chem 53: 9342–9347 16302745 10.1021/jf051488w

[bib48] Phipps WR, Martini MC, Lampe JW, Slavin JL, Kurzer MS (1993) Effect of flax seed ingestion on the menstrual cycle. J Clin Endocrinol Metab 77: 1215–1219 8077314 10.1210/jcem.77.5.8077314

[bib49] Pietinen P, Stumpf K, Männistö S, Kataja V, Uusitupa M, Adlercreutz H (2001) Serum enterolactone and risk of breast cancer: a case-control study in eastern Finland. Cancer Epidemiol Biomarkers Prev 10: 339–344 11319174

[bib50] Piller R, Chang-Claude J, Linseisen J (2006a) Plasma enterolactone and genistein and the risk of premenopausal breast cancer. Eur J Cancer Prev 15: 225–232 16679865 10.1097/01.cej.0000197449.56862.75

[bib51] Piller R, Verla-Tebit E, Wang-Gohrke S, Linseisen J, Chang-Claude J (2006b) CYP17 genotype modifies the association between lignan supply and premenopausal breast cancer risk in humans. J Nutr 136: 1596–1603 16702327 10.1093/jn/136.6.1596

[bib52] Pousette A, Gustafsson SA, Thornblad AM, Nordgren A, Sallstrom J, Lindgren A, Sundelin P, Gustafsson JA (1986) Quantitation of estrogen receptor in seventy-five specimens of breast cancer: comparison between an immunoassay (Abbott ER-EIA monoclonal) and a [3H]estradiol binding assay based on isoelectric focusing in polyacrylamide gel. Cancer Res 46: 4308s–4309s 3524812

[bib53] Prasad K (2000) Antioxidant activity of secoisolariciresinol diglucoside-derived metabolites, secoisolariciresinol, enterodiol, and enterolactone. Int J Angiol 9: 220–225 11062311 10.1007/BF01623898

[bib54] Rickard SE, Yuan YV, Thompson LU (2000) Plasma insulin-like growth factor I levels in rats are reduced by dietary supplementation of flaxseed or its lignan secoisolariciresinol diglycoside. Cancer Lett 161: 47–55 11078912 10.1016/s0304-3835(00)00592-9

[bib55] Schwartz H, Sontag G (2006) Determination of secoisolariciresinol, lariciresinol and isolariciresinol in plant foods by high performance liquid chromatography coupled with coulometric electrode array detection. J Chromatogr B Analyt Technol Biomed Life Sci 838: 78–85 10.1016/j.jchromb.2006.03.058 16750660

[bib56] Serraino M, Thompson LU (1991) The effect of flaxseed supplementation on early risk markers for mammary carcinogenesis. Cancer Lett 60: 135–142 1657368 10.1016/0304-3835(91)90220-c

[bib57] Serraino M, Thompson LU (1992) The effect of flaxseed supplementation on the initiation and promotional stages of mammary tumorigenesis. Nutr Cancer 17: 153–159 1584708 10.1080/01635589209514182

[bib58] Suzuki R, Rylander-Rudqvist T, Ye W, Saji S, Adlercreutz H, Wolk A (2008) Dietary fiber intake and risk of postmenopausal breast cancer defined by estrogen and progesterone receptor status-A prospective cohort study among Swedish women. Int J Cancer 122: 403–412 17764112 10.1002/ijc.23060

[bib59] Suzuki R, Rylander-Rudqvist T, Ye W, Saji S, Wolk A (2006) Body weight and postmenopausal breast cancer risk defined by estrogen and progesterone receptor status among Swedish women: A Prospective Cohort Study. Int J Cancer 119: 1683–1689 16646051 10.1002/ijc.22034

[bib60] Thompson LU, Boucher BA, Liu Z, Cotterchio M, Kreiger N (2006) Phytoestrogen content of foods consumed in Canada, including isoflavones, lignans, and coumestan. Nutr Cancer 54: 184–201 16898863 10.1207/s15327914nc5402_5

[bib61] Thompson LU, Chen JM, Li T, Strasser-Weippl K, Goss PE (2005) Dietary flaxseed alters tumor biological markers in postmenopausal breast cancer. Clin Cancer Res 11: 3828–3835 15897583 10.1158/1078-0432.CCR-04-2326

[bib62] Touillaud MS, Thiebaut AC, Fournier A, Niravong M, Boutron-Ruault MC, Clavel-Chapelon F (2007) Dietary lignan intake and postmenopausal breast cancer risk by estrogen and progesterone receptor status. J Natl Cancer Inst 99: 475–486 17374837 10.1093/jnci/djk096 PMC2292813

[bib63] Valsta LM, Kilkkinen A, Mazur W, Nurmi T, Lampi AM, Ovaskainen ML, Korhonen T, Adlercreutz H, Pietinen P (2003) Phyto-oestrogen database of foods and average intake in Finland. Br J Nutr 89(Suppl 1): S31–S38 12725654 10.1079/BJN2002794

[bib64] Verheus M, van Gils CH, Keinan-Boker L, Grace PB, Bingham SA, Peeters PH (2007) Plasma phytoestrogens and subsequent breast cancer risk. J Clin Oncol 25: 648–655 17200150 10.1200/JCO.2006.06.0244

[bib65] Welshons WV, Murphy CS, Koch R, Calaf G, Jordan VC (1987) Stimulation of breast cancer cells *in vitro* by the environmental estrogen enterolactone and the phytoestrogen equol. Breast Cancer Res Treat 10: 169–175 3427225 10.1007/BF01810580

[bib66] Wolk A, Bergstrom R, Hunter D, Willett W, Ljung H, Holmberg L, Bergkvist L, Bruce A, Adami HO (1998) A prospective study of association of monounsaturated fat and other types of fat with risk of breast cancer. Arch Intern Med 158: 41–45 9437377 10.1001/archinte.158.1.41

